# A Stochastic Filling and Modeling Algorithm of Non-Equal Diameter Particles with Specified Probability Density for Porous Permeable Materials

**DOI:** 10.3390/ma15144733

**Published:** 2022-07-06

**Authors:** Wei Zhang, Lile He, Fazhan Wang, Guangyong Zhang

**Affiliations:** 1 School of Mechanical and Electrical Engineering, Xi’an University of Architecture and Technology, Xi’an 710055, China; zhangweixjd@xauat.edu.cn (W.Z.); hllnh2013@163.com (L.H.); 2 State Key Laboratory of Metal Extrusion and Forging Equipment Technology, China National Heavy Machinery Research Institute Co., Ltd., Xi’an 710018, China; zhanggy137@163.com

**Keywords:** non-equal diameter particles, specified probability density, random filling, permeability, porous materials

## Abstract

In this paper, a model generation algorithm for non-equal diameter particles with a specified probability density distribution is proposed. Based on considering the randomness of the size and distribution of the particles, the compact stacking of the particles is realized by the compactness algorithm, and then the spatial distribution of the tightly compacted particles is made to meet the random distribution of the specified probability density and the specified volume fraction by the filtering algorithm. The computational efficiency and effectiveness of the algorithm are verified, and the effects of the particle size and volume fraction on the distribution are analyzed. Finally, the proposed model has been used to study the permeability of a titanium porous filter cartridge. The results show that the size and location of the particle samples that are generated by the proposed algorithm follow specified probability distributions according to the requirements, and the volume fraction can be adjusted. Compared with the traditional algorithm, the computational effort and complexity are reduced. The resultant model can be used to study the permeability of porous materials and provide modeling support for structural optimization and further simulation of porous materials.

## 1. Introduction

Many new functional materials are bonded and compressed by granular materials with a pore structure that follows a certain probability distribution, such as titanium porous filter cartridge and metal-matrix composites. In order to obtain a mesoscopic mechanism for materials with a specific random distribution of particles, it is necessary to study the relationship between the properties of granular materials and the microstructure morphological characteristics of the formed materials, so as to carry out an effective structural performance design of the material [1,2]. However, because of the complex mesoscopic pore structure, it is difficult to realize the visualization of mesoscopic mechanism, for example, the seepage properties, through experiments [3]. Therefore, a meso-model with a specified probability density distribution is built to provide reference for improving the accuracy and efficiency of porous media meso-modeling.

As for the generation algorithm of particle distribution, Primera et al. [4] proposed the method of triangulation to describe the pore size, but this method was completed through two-dimensional slices and could not fully represent the three-dimensional morphology of the pores. Tory E M et al. [5] continuously filled the fixed space with the sequence addition method and completed the sequence filling of the powder layer based on considering the stability of the powder particles and the interaction force between the particles. Jodrey W S et al. [6] used the non-sequence rearrangement algorithm to start position allocation with large-diameter particles first, allocating the position with small-diameter particles, and then reducing the overlap degree by moving particles. Cundall P A et al. [7] made the stacking process of particles similar to the flow process of dynamic fluid based on the discrete element method and realized the stacking of powder particles. A. Bezrukov et al. [8] describes two algorithms for the generation of random packings of spheres with arbitrary diameter distribution. The first algorithm is the force-biased algorithm. It produces isotropic packings of a very high density. The second algorithm is the Jodrey–Tory sedimentation algorithm, which simulates the successive packing of a container with spheres following gravitation. It yields packings of a lower density and of weak anisotropy. Although the above algorithms could achieve the stacking of powder particles, there existed some shortcomings in the randomness of spatial distribution, and the filling efficiency was low. At the same time, the above algorithms inevitably need to constantly detect the interference between particles, which means tedious calculation.

In addition, Bailakanavar M et al. [9] used the random order adsorption algorithm to determine whether the current particles interfered with the existing particles, retained the data of the non-interfered particles, and then established the relevant model. Xin Zhenyang et al. [10] used the perturbation algorithm to assign regular distribution positions to all particles and applied the perturbation to the amount of random distance between each particle. Through interference judgment, the position information of particles was retained. Note that through the above, the generation algorithms could ensure the randomness of the space to a certain extent. Due to the need to compare the position relationship between the specified particles and all the particles in the distribution process, the computation burden is somewhat high, which affects the efficiency of the method.

The transmission characteristics of spherical packing include electrical conductivity and permeability, among which permeability is one of the most studied properties [11,12,13]. Experimental and theoretical studies on permeability have achieved certain results, such as Darcy’s Rule for fluid flow in porous media and the empirical formula Kozeny relation for permeability [14]. As the pore structure of sphere accumulation is complex like that of other porous media, it is difficult to study fluid flow in porous media by traditional methods [15].

To solve the above problems, this paper proposes a method to generate the meso-model of non-equal diameter particles with a specified probability density distribution. The filling method of particles avoids the tedious calculation that is caused by interference detection, and since the movement of particles is only related to a small number of particles in the neighborhood, the modeling efficiency is further improved to a significant degree. Through the comparison of computation with the traditional methods and a hypothesis test, the efficiency and effectiveness of the algorithm are verified, illustrating that the proposed generation algorithm could effectively build a meso-model for porous media and titanium foam with a specified probability density distribution. The lattice Boltzmann method is used to calculate the permeability of porous material with different probability density distributions. The results show that the porous material meso-model that is established by the proposed algorithm in this paper can provide a basis for further numerical simulation of fluid permeability.

## 2. Generation Algorithm for Meso-Distribution Particles

Considering the randomness of the particles in size and distribution, Python is employed to build the algorithm, and gives the generation steps of the particles a meso-model, according to the specified probability density. The proposed algorithm consists of two parts. The first part is the compactness algorithm, which causes the particles with the size of a specified probability density distribution to stack closely together. The second part is the filtering algorithm. By using this method, the distribution of the particles in the spatial position according to the specified probability density can be realized, and the specified volume fraction can be achieved based on the requirements.

### 2.1. Compactness Algorithm for Particles

Let i represent the direction of the x axis, j represent the direction of the y axis, and k represent the direction of the z axis, respectively, corresponding to columns, rows and layers. The particles of random sizes are generated in the cube area according to the direction of the column, row and layer successively, and the tight compactness is realized by adjusting the position between the particles.

#### 2.1.1. Formation of Non-Equal Diameter Particles

According to the actual demand, the particle size is randomly distributed according to the specified probability density by defining an appropriate sphere diameter range. 

The expectation of X_r is

$$E\left(X\_r\right)=\int_{A}^{}\mu fd\mu$$

In practice, the algorithm generates particles with a radius that is randomly distributed on the interval [*a*, *b*]; the average of the radius is

$$r_{avg}=E\left(X\_r\right)=\int_{a}^{b}xf\left(x\right)dx$$

#### 2.1.2. Determine Initial Position

At this step, the algorithm places the random sized particles roughly in a 3D matrix pattern, with particle positions vibrated by random noises. To place the first particle with radius $r_{1\;}$near coordinates (0, 0, 0), the algorithm considers the particle radius, the cube area boundary restriction and the random noise. The resulting center coordinates of the particle are noted as $\left(x_{1},\;y_{1},\;z_{1}\right)$, where $x_{1}=r_{1}+d_{noise}$*,* $y_{1}=r_{1}+d_{noise}\;$and$\;z_{1}=r_{1}+d_{noise}$*;* the second particle with radius $r_{2}$ is placed at coordinates $\left(x_{2},\;y_{2},\;z_{2}\right)$, where $x_{2}=x_{1}+r_{1}+r_{2}+d_{noise}$, $y_{2}=r_{2}+d_{noise}$, $z_{2}=r_{2}+d_{noise}$. the third particle with radius $r_{3}$ is placed at $\left(x_{2}+r_{2}+r_{3}+d_{noise},\;r_{3}+d_{noise},\;r_{3}+d_{noise}\right)$, etc. The notation $d_{noise}$ stands for the random noise that the algorithm introduces; it takes a different value each time it appears. The particle placement process continues until the first row on the xy-plane is filled. Next, the algorithm places the second row close to the first row on the xy-plane and the row placement process repeats until the first xy-layer is filled. Afterwards, the second particle layer is placed closely above the first layer and the layer placement process repeats until there are enough layers. Prior to the placement of any particles, the algorithm estimates the number of particles to generate by considering the scenario where the cube area is filled with particles of equal radius $r_{avg}$. Assume the length of the edges of the cube along the x,y,z-axis are$\;L_{x},L_{y}$ and $L_{z}$, respectively; the number of particles in each row, the number of rows in each layer and the number of layers are estimated as $N_{x}=L_{x}/\left(2r_{avg}+\rho \right)$, $\;N_{y}=L_{y}/\left(2r_{avg}+\rho \right)$ and $N_{z}=L_{z}/\left(2r_{avg}+\rho \right)$, respectively. The notation ρ stands for the upper bound of the gap distance between adjacent particles and is enforced by the compactness step (Section 2.1.3). Since the density of the particles is increased by the compactness step and the goal is to distribute the particles in the entire cube area following a given probability density, the estimated values $N_{x},\;N_{y}$ and $N_{z}$ are further scaled up by an overfill factor $f_{ov}$ > 1. After the compactness step, the excessive particles that remain outside of the cube boundaries are easily pruned. 

**Figure 1:**
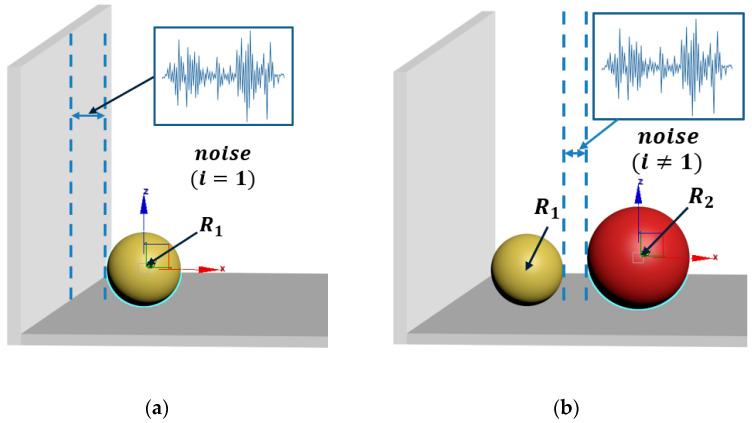
Principle of initial position determination. (**a**) The position of new particle generated (*i* = 1). (**b**) The position of new particle generated (*i* ≠ 1).

To ensure that each newly generated particle and the last generated particle are adjacent in the same row and layer, the adjacent scales vibrate within a very small range. The advantage of this method is that the detection of particle interference can be omitted, and thus the randomness of the position can be retained, which significantly simplifies the computational effort of the algorithm. The logical steps are as follows:

(1) By specifying a minimal disturbance init_noise to add a segment of white noise, its power spectral density will be constant in the whole frequency domain, as shown in Figure 1 (when i=1, the *x* coordinate of the center is evaluated on [x_min,x_min+init_noise] in a uniformly distributed scale, where *x_min* is simply the radius of the particle and *init_noise* is a preselected constant to control the amplitude of the noise). Otherwise, make the minimum coordinate x_min of the newly generated particles in row j of the kth layer equal to the previous maximum x coordinate prev_x_max, plus the radius of the new particle, before taking the corresponding disturbance distance.

(2) Similarly, when j=1, the y coordinate of the center is evaluated at a uniformly distributed scale on [y_min,y_min+init_noise], where y_min is simply the particle radius. Otherwise, since the (j−1)th row particles in layer k have been determined, the minimum coordinate y_min of the newly generated particles in layer k should be equal to the maximum y coordinate prev_y_max of the (j−1)th row, plus the radius of the new particle, before taking the corresponding disturbance distance.

(3) Similarly, when k=1, the z coordinate of the center is evaluated on [z_min,z_min+init_noise], where *z_min* is simply the radius of the particle. Otherwise, since the particles at the (k−1)th layer have been determined, the minimum coordinate z_min of the newly generated particles should be equal to the maximum *z* coordinate prev_z_max (k−1)th layer, plus the radius of the new particle, before taking the corresponding disturbance distance.

**Figure 2:**
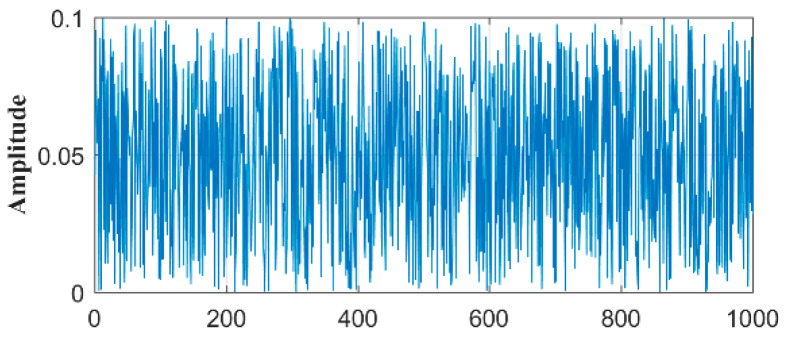
White noise waveform.

If the amplitude of the noise is 0.1 and the mean value is 0.05, when the new particle has been generated 1000 times, the graph of the noise can be obtained, as shown in Figure 2.

The pseudocode of the placement process is as follows in Algorithm 1.
**Algorithm 1** Algorithm of initial position determination **for** k in [1, fov*Nz]   z_max = −∞   **for** j in [1, fov*Ny]     y_max = −∞     prev_x_max = −∞     **for** i in [1, fov*Nx]       generate particle with random size r       **if** i = 1 then         x_min = r       **else**         x_min = prev_x_max + r       **if** j = 1 then         y_min = r       **else**         y_min = prev_y_max + r       **if** k = 1 then         z_min = r       **else**         z_min = prev_z_max + r       x_new = u(x_min, x_min+init_noise)       y_new = u(y_min, y_min+init_noise)       y_new = u(z_min, z_min+init_noise)       place the newly generated particle at (x_new, y_new, z_new)       prev_x_max = max(prev_x_max, x_new+r)       y_max = max(y_max, y_new+r)       z_max = max(z_max, z_new+r)     prev_y_max = y_max   prev_z_max = z_max

In the pseudo code, u (*a*, *b*) generates a uniformly distributed random value on the interval [*a*, *b*].

#### 2.1.3. Compactness of Particles

Assume that the randomly generated particles are stored in a 3-dimensional array M, whose size is $I_{max}\times J_{max}\times K_{max}$, $n_{neighbor}$ is a small positive integer, and ρ is a small positive value. The position of the particles will be repositioned by coordinate translation.

By calculating the distance dz between the particle and the $n_{neighbor}$ neighboring particles in layer (k−1), local neighborhood compression can be achieved to speed up the iterative process and more tightly compact the particles. While dy is the distance between the particles and the particles in the $n_{neighbor}$ range that are located in row (j−1) of layer k, dx is the distance between the particle and the particles in the $n_{neighbor}$ range that are located in row j, column (i−1)  of layer k. Let dw=Max (dx, dy,dz)(w∈ x, y, z). Move the particle by dw/3 in the w direction. Repeat this step until dx, dy,dz<ρ.

The center coordinate of the particle P=(x,y,z) is reduced by 1/3dw, dw=Max (dx, dy,dz)(w∈ x,y,z), and a new position $P\;{}^{\prime }=\left(x{}^{\prime },y\;{}^{\prime },z\;{}^{\prime }\right)\;$ is obtained. The coordinates of the new position are derived as follows.

$$\left[\begin{array}{c} x{}^{\prime }\\ y{}^{\prime }\\ \begin{array}{c} z{}^{\prime }\\ 1 \end{array} \end{array}\right]=\left[\begin{array}{cc} \begin{array}{ccc} -1 & 0 & 0\\ 0 & -1 & 0\\ \begin{array}{c} 0\\ 0 \end{array} & \begin{array}{c} 0\\ 0 \end{array} & \begin{array}{c} -1\\ 0 \end{array} \end{array} & \begin{array}{c} x\\ y\\ \begin{array}{c} z\\ 1 \end{array} \end{array} \end{array}\right]\cdot \frac{1}{3}\left[\begin{array}{c} dw\_x\\ dw\_y\\ \begin{array}{c} dw\_z\\ 3 \end{array} \end{array}\right]$$

According to the aforementioned methods, the particles at the initial position can be further compressed in distance to be tightly compacted.

The pseudocode of the compactness process is as follows in Algorithm 2.
**Algorithm 2** Compactness algorithm**for***k* **in** [0, *K_max_*−1]   **for**
*j* **in** [0, *J_max_*−1]     **for**
*i* **in** [0, *I_max_*−1]       *x_boundary_* = max (*x*|*x* is the x coordinate of points on the particles at *M* [*i*−1,*j*,*k*])        *y_boundary_* = max (*y*|*y* is the y coordinate of points on the particles at *M* [*i*,*j_n_*,*k*],            where *j_n_* in [*j*−*n_neighbor_*, *j*+*n_neighbor_*])       *z_boundary_* = max (*z*|*z* is the z coordinate of points on the particles at *M* [*i*,*j_n_*,*k_n_*],            where *j_n_* in [*j*−*n_neighbor_*, *j*+*n_neighbor_*] and *k_n_* in [*k*−*n_neighbor_*, *k*+*n_neighbor_*]    Assume:     Plane *P_x_* parallel to the *yz*-plane intersects the *x*-axis at *x_boundary_*.     Plane *P_y_* parallel to the *xz*-plane intersects the *y*-axis at *y_boundary_*.     Plane *P_z_* parallel to the *xy*-plane intersects the *z*-axis at *z_boundary_*.        *d_x_* = distance between *M* [*i,j,k*] and *P_x_*        *d_y_* = distance between *M* [*i,j,k*] and *P_y_*        *d_z_* = distance between *M* [*i,j,k*] and *P_z_*         **while** max (*d_x_, d_y_, d_z_*) ⩾ ρ           **if**
*d_x_* = max (*d_x_, d_y_, d_z_*)             subtract *d_x_*/3 from the x coordinate of *M* [*i,j*,*k*]           **else if** *d_y_* = max (*d_x_, d_y_, d_z_*)             subtract *d_y_*/3 from the y coordinate of *M* [*i,j*,*k*]           **else** *d_z_* = max (*d_x_, d_y_, d_z_*)             subtract *d_z_*/3 from the z coordinate of *M* [*i,j*,*k*]           Recalculate *d_x_,d_y_,d_z_*           **if** loop has executed more than 1000 times:           Report exception and quit 

**Figure 3:**
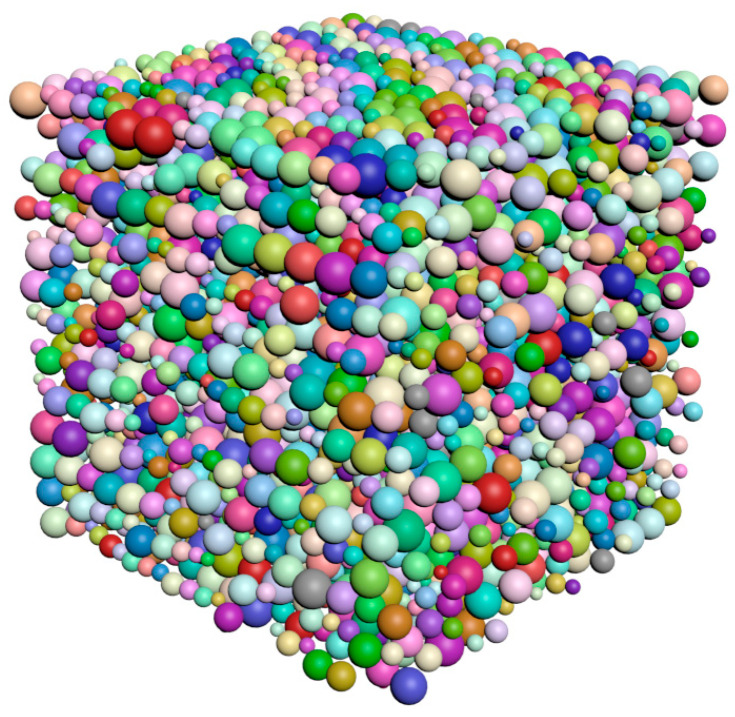
Results of compactness algorithm execution.

Figure 3 is the compactness model diagram that is formed after the implementation of the compactness algorithm. The particles whose size complies with the specified distribution law are packed tightly in the cube region. 

### 2.2. Filtering Algorithm

After the compactness algorithm, the particles with a random size distribution can be tightly compacted and a higher volume fraction of bulk density can be obtained. However, the spatial distribution cannot meet the random distribution of the assigned probability density, and the volume fraction cannot be adjusted according to the demand. Filtering algorithms help with this process. This paper makes the following assumptions:

(1) Assuming that the volume of the cube in which the particles are located is v, such that the particles are uniformly distributed, the probability density is the same throughout the cube;

$$\;u_{x,\;y,\;z}=1/v$$

(2) After the execution of the compactness algorithm and before the execution of the filtering algorithm, the total volume of the particles is $v_{g}$;

(3) If the target volume fraction is α, the total volume of the particles corresponding to this volume fraction is αv;

(4) The target probability density function is $f_{x,y,z}$.

Given that a small zone with volume dv is centered at (x,y,z) in the cube, the probability of retaining the particles near (x,y,z) by the filtering algorithm is

$$s_{x,y,z}=\frac{f_{x,y,z}\alpha \;vdv}{u_{x,y,z}v_{g}dv}$$

Substitute the hypothesis, simplify it, and we can achieve

$$s_{x,y,z}=\frac{\alpha \;v^{2}}{v_{g}}f_{x,y,z}$$

Assuming that the tightly packed particles are stored in a three-dimensional array $I_{max}\times J_{max}\times K_{max}$, after using the aforementioned methods, the particles that do not conform to the given probability density distribution in space are filtered out.

The pseudocode is as follows in Algorithm 3.
**Algorithm 3** Filtering algorithm**for***k* **in** [0, *K_max_−*1]  **for**
*j* **in** [0, *J_max_−*1]   **for**
*I* **in** [0, *I_max_−*1]    Let *x,y,z* be the coordinates of the object *M* [*i,j,k*]    **if** s > α*v^2^*/*v_g_*∙∙*f_x,y,z_*    Delete M [i,j,k]

**Figure 4:**
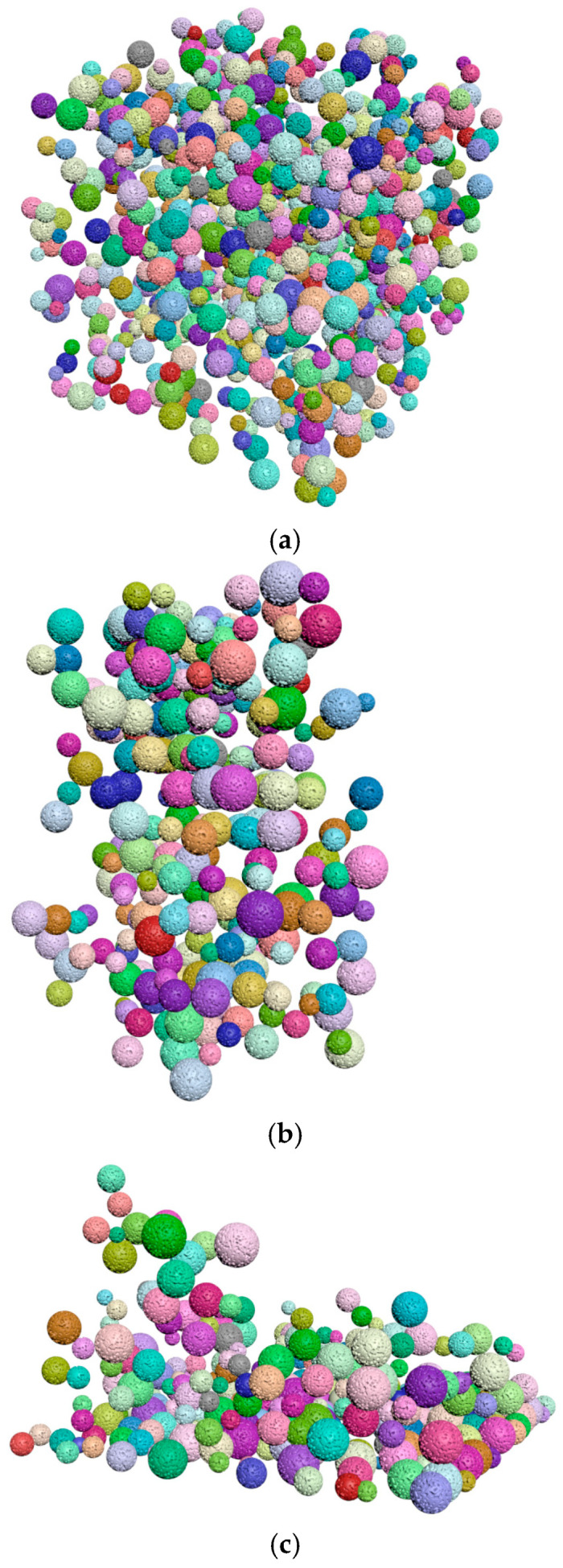
The result of the filtering algorithm execution. (**a**) Uniform distribution along x, y, and z, (**b**) Normal distribution along the x direction, (**c**) Exponential distribution along the z direction.

Figure 4 shows examples of common probability density functions (uniform, normal and exponential), as generated by the filtering algorithm.

## 3. Influence of Parameters on the Algorithm

The parameters of the algorithm directly affect its performance. In this section, the influence of the design parameters on particle filling and distribution effect in the application of this algorithm are discussed. The design parameters mainly include the particle size and the volume fraction. This section presents the distribution and filling effect and summarizes the related influence.

### 3.1. Distribution of Particles with Different Size Ranges with Same Probability Density

Taking the uniform distribution in the x, y and z directions as an example, the distribution of the particles with different size ranges is simulated by using the proposed algorithm. The volume fraction is 5%, while the volume of the cube is 125 mm. The total volume of the particles is equal. The distribution results are shown in Table 1.

As can be seen from Table 1, when the volume fraction remains unchanged, the number of particles decreases significantly with the increase in particle size, and the distribution becomes sparser.

### 3.2. Particles Distribution with Same Probability Density and Different Volume Fraction

In this section, the uniform distribution in the x, y and z directions is taken as an example to simulate the distribution of particles in different volume fraction ranges by using the proposed algorithm. The radius sizes of the particles are all between 0.15–0.25 mm. The total volume of the cube is 125 mm^3^. The distribution results are shown in Table 2.

As can be seen from Table 2, when the radius size of the particles remains unchanged, the volume of the particles increases with the increase in volume fraction, but the maximum volume fraction of the particles cannot exceed the volume fraction of the tightest compactness that is formed by the compactness algorithm.

## 4. Algorithm Analysis

The effectiveness of the algorithm is analyzed in two regards, namely, the efficiency and the randomness. First, the efficiency of the algorithm is demonstrated from the perspective of computational complexity. Secondly, the sample that is generated by the algorithm is used to verify the randomness of the random algorithm in particle size and location by the statistical hypothesis testing method.

### 4.1. Computational Efficiency

Taking the uniform distribution as an example, it is assumed that n particles obey the random distribution, and the efficiency analysis of the take-and-place algorithm and the algorithm proposed in this paper is as follows:

(1) The major steps of the take-and-place algorithm are as follows: randomly generate the position of the new particle and determine whether there is any interference between the current particle and the existing particles. If there is no interference, the relevant data of the current particle are saved, and the particle is accepted; otherwise, the algorithm needs to try new locations again and again. Therefore, the take-and-place algorithm not only reduces the randomness of sample generation, but also comes with the computational complexity of$\;c_{1}\;\left(m_{1}+2m_{2}+,\ldots +\left(k-1\right)m_{k}+,\ldots +\left(n-1\right)m_{n}\right)$ that is far greater than $O\;\left(n^{2}\right)$. To clarify, $m_{k}$ is the number of repeated generation positions when filling the kth particle and a positive constant $c_{1}$ is independent of n;

(2) As mentioned in Section 2, the algorithm that is proposed in this paper consists of two parts. In the first part of the algorithm, the position of each particle is only related to a small number of particles in its neighborhood when the initial position is generated or the tightness of the particles is adjusted. Therefore, the computational complexity is $c_{1}\;{}^{\prime }n_{1}$, where$\;n_{1}$ is the number of particles and a positive constant $c_{1}{}^{\prime }$ is independent of $n_{1}$. In the second part of the algorithm, each particle is independently processed, and the complexity is$\;c_{2}\;{}^{\prime }n_{1}$, where$\;c_{2}{}^{\prime }$ is a constant independent of $n_{1}$. Accordingly, the overall computational complexity of our algorithm is O(n).

The computational complexity of the particle random generation algorithm that is proposed in this paper is significantly lower than that of the traditional algorithm, and it is highly efficient.

### 4.2. The Randomness of Generated Samples

#### 4.2.1. Random Test of Particles Size

**Figure 5:**
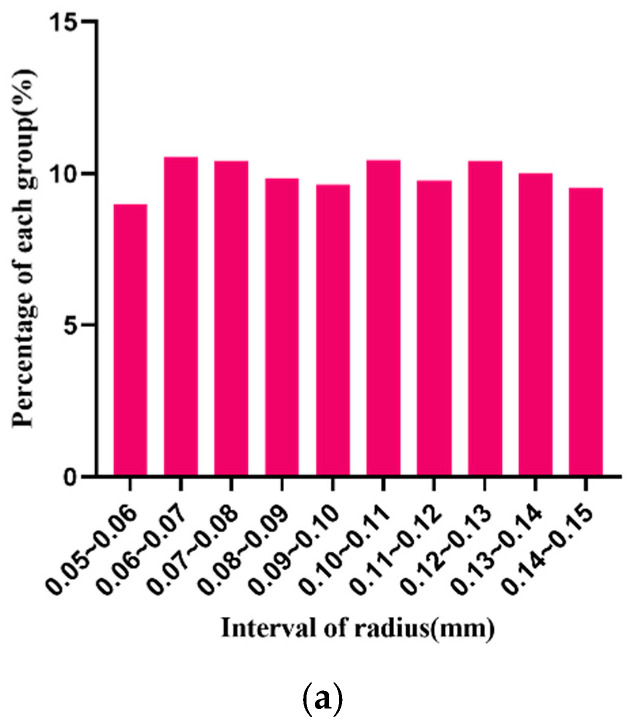

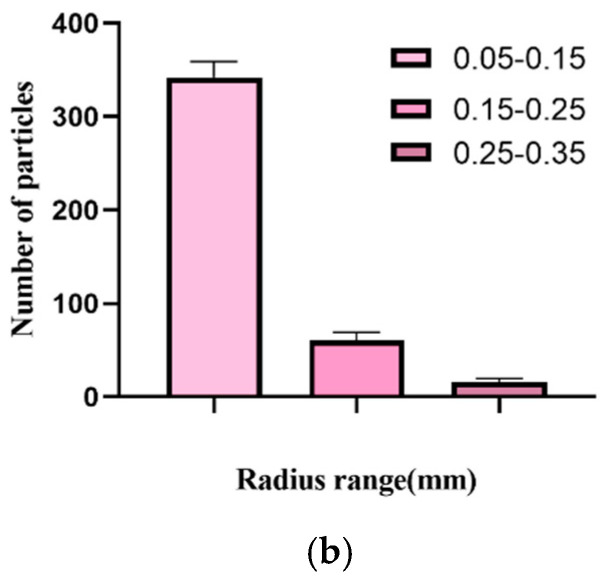
Distribution of particle radius in each interval. (**a**) Percentage of particles in each radius, (**b**) The number and standard deviation of particles within each radius.

At present, most of the simulations focus on the case of uniform and random distribution, so the random characteristics of the samples with uniform and random distribution are studied, and an $\chi^{2}$ test [16] is used to evaluate the uniformity of the samples. The statistic test formula is

$$\chi^{2}=\frac{m}{\epsilon }\overset{m}{\underset{i=1}{\sum }}{\left(\epsilon_{i}-\frac{\epsilon }{m}\right)}^{2}$$

where ϵ is the number of all random numbers, m is the number of intervals, and $\epsilon_{i}$ is the number of the i th interval. In the uniformity test of the algorithm, we generated 3485 samples whose radii are on the interval (0.05, 0.15). The percentages of each radius are shown in Figure 5.

The particles sizes are evenly distributed in each interval. Assuming that the degree of freedom is 9, the calculated $\chi^{2}$ value of radius R is 11.72, and the asymptotic significance is 0.230, greater than 0.05. According to statistic theory, the $\chi^{2}$ distribution table shows that the distribution of particle radius R that is obtained by the algorithm can be considered as an ideal uniform random distribution. The test statistical scale is shown in Table 3.

#### 4.2.2. Random Test of Particle Position

Taking normal distribution as an example, the Shapiro–Wilk test method [17] and the Kolmogorov–Smirnov test method [18,19] are used to test the normality of the samples that are generated by the random algorithms, using the compactness algorithm to make the spherical center coordinates of 301 particles normally distributed along the x-axis. Table 4 is the analysis result of the normality test.

**Figure 6:**
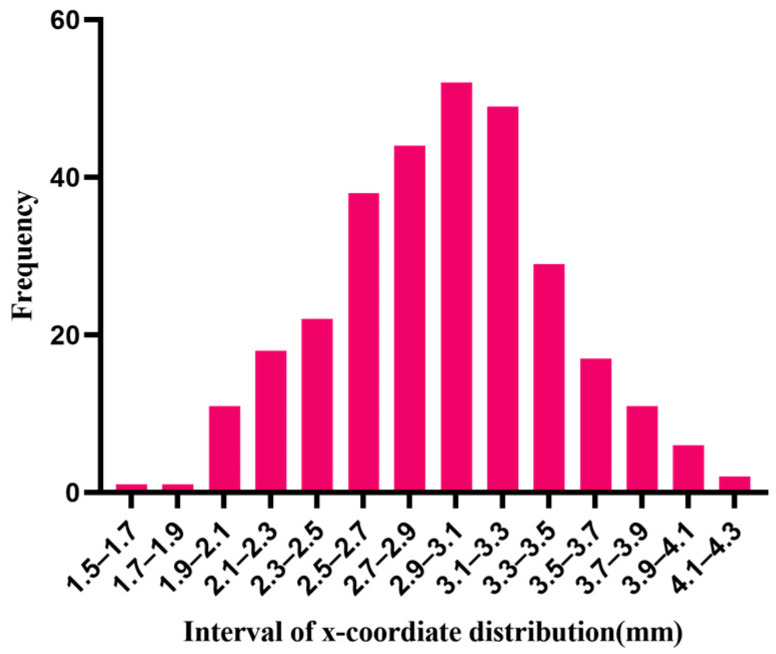
Particle coordinate distribution along the x direction.

As can be seen from Table 4, the sample data did not show statistical significance (*p* > 0.05), which means that the hypothesis (hypothesis: the data are normally distributed) is accepted and the sample has the characteristic of normality. The histogram of the sample distribution along the x-axis is shown in Figure 6.

## 5. Permeability of Porous Media

Titanium porous filter cartridge is made of porous titanium metal filter material by the powder metallurgy method. Its internal pores are curved and crisscross, and the filtering mechanism is typical deep filtration. Permeability is the ability of a porous medium to allow fluid matter to pass through. In this section, the lattice Boltzmann method is used to study the flow of fluid under the condition of mass particle filling and the influence of initial particle distribution on fluid permeability by using the meso-model of porous media that is constructed by the above algorithm.

### 5.1. Lattice Boltzmann Method

The motion of a fluid can be described by a set of partial differential equations, such as the Navier–Stokes equations, which are highly nonlinear in most cases and find it very difficult to obtain analytical solutions. The Lattice Boltzmann method is used to solve the numerical solution of the fluid motion equation by means of the discrete method. The lattice Boltzmann method can be regarded as a special discrete scheme of a continuous Boltzmann equation, as shown in the following formula.

$$g_{i}\left(x+e_{i}\delta_{t},t+\delta_{t}\right)-g_{i}\left(x,t\right)=\Omega_{i}\left(x,t\right)$$

where g is the discrete distribution function, e is the velocity space, i is the type of velocity, $\delta_{t}$ is the discrete time step, t is the current time step, x is a point on the grid, and Ω is the change caused by collision.

According to the operator that is proposed by Bhatnagar, Gross, and Krook [20], $\Omega_{i}\left(x,t\right)\;$can be replaced, as shown in the following formula.

$$g_{i}\left(x+e_{i}\delta_{t},t+\delta_{t}\right)-g_{i}\left(x,t\right)=\frac{1}{\tau }\left(g_{i}^{eq}-g_{i}\right)$$

where $g_{i}^{eq}$ is an equilibrium distribution function to be determined; τ is the relaxation time. 

**Figure 7:**
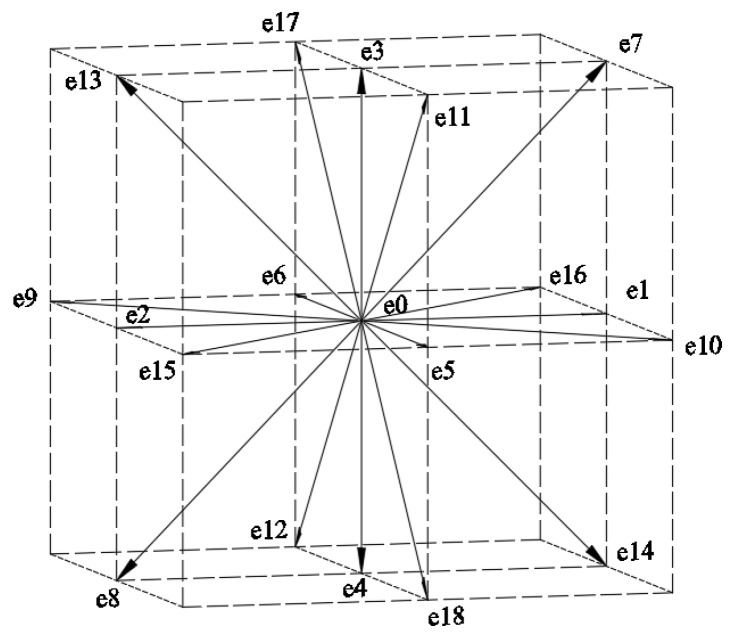
Discrete velocity diagram of lattice points.

The $D_{d}Q_{m}$ model that is proposed by Qian et al. [21] is the basic model of the lattice Boltzmann model, where d represents d-dimensional space and m represents the number of discrete velocities of the lattice. In this paper, the velocity is discretized into 19 directions in three-dimensional space, as shown in Figure 7.

The discrete component of the velocity is

$$E=\left[e_{0},e_{1},e_{2},e_{3},e_{4},e_{5},e_{6},e_{7},\cdots ,e_{18}\right]$$

The equilibrium equation [21] of this model is

$$g_{i}^{eq}=\rho_{m}\omega_{i}\left[1+\frac{e_{i}\cdot \mu }{c_{s}^{2}}+\frac{{\left(e_{i}\cdot \hat{\sigma }\right)}^{2}}{2c_{s}^{4}}-\frac{{\hat{\sigma }}^{2}}{2c_{s}^{2}}\right]\;i=0,1,2\cdots ,18$$

where $C_{s}$ is the lattice speed, ω is the weight coefficient, $\hat{\sigma }$ is the fluid velocity, and $\rho_{m}$ is the fluid density. The lattice velocity $C_{s}$ is as follows.

$$C_{s}=\frac{c}{\sqrt{3}}$$

where c is the ratio of grid step to time step, and its value is 1. The calculation of $\omega_{i}$ is shown in the following formula.

$$\omega_{i}=\{\begin{array}{c} 1/3\\ 1/18\\ 1/36 \end{array}\begin{array}{c} e_{i}^{2}=0\\ e_{i}^{2}=c^{2}\\ e_{i}^{2}=2c^{2} \end{array}$$

Let $g_{i}\left(x_{t},t\right)\;$be the distribution function of time t, at lattice point x, velocity e, then the evolution equation of the distribution function is

$$g_{i}\left(x+e_{i}\delta_{t},t+\delta_{t}\right)-g_{i}\left(x,t\right)\;=\frac{1}{\tau }\left(g_{i}^{eq}\left(x_{t},t\right)-g_{i}\left(x_{t},t\right)\right)$$

### 5.2. Darcy’s Law

When single-phase flow flows in porous media at a low Reynolds number, it follows Darcy’s Law, also known as the Darcy model, which is one of the most basic and commonly used mathematical models for macroscopic seepage, as shown in the following equation

$$\sigma =-\frac{h}{\nu }\nabla p_{l}$$

where σ is the Darcy velocity, h  is the permeability of porous media, ν is the universality coefficient of the fluid, $p_{l}$ is the fluid pressure, and ∇  is the Hamiltonian operator. 

According to the distribution function, the macroscopic parameters of the fluid density $\rho_{l}$ and fluid velocity σ can be obtained from formula

$$\rho_{l}=\underset{i}{\sum }g_{i}\;\sigma =\frac{1}{\rho_{l}}\underset{i}{\sum }g_{i}\cdot e_{i}$$

The permeability of porous media can be calculated according to Darcy’s formula [22].

$$h=\frac{\nu \bar{\sigma }}{∆p_{l}}$$

where h is the permeability of the porous media, ν is the coefficient of motion universality, $∆p_{l}$ is the pressure difference, and $\bar{\sigma }$ is the average speed.

### 5.3. Lattice Boltzmann Method Simulation Procedures

The lattice Boltzmann method simulation program structure is collision-migration. The specific process is as follows:

(1) Setting of initial conditions;
(2) Execute collision at time t;
(3) Boundary processing;
(4) Calculate macroscopic quantities;
(5) Check whether convergence exists. If not, return to Step 2. Otherwise, go to the next step;
(6) Output the result.

**Figure 8:**
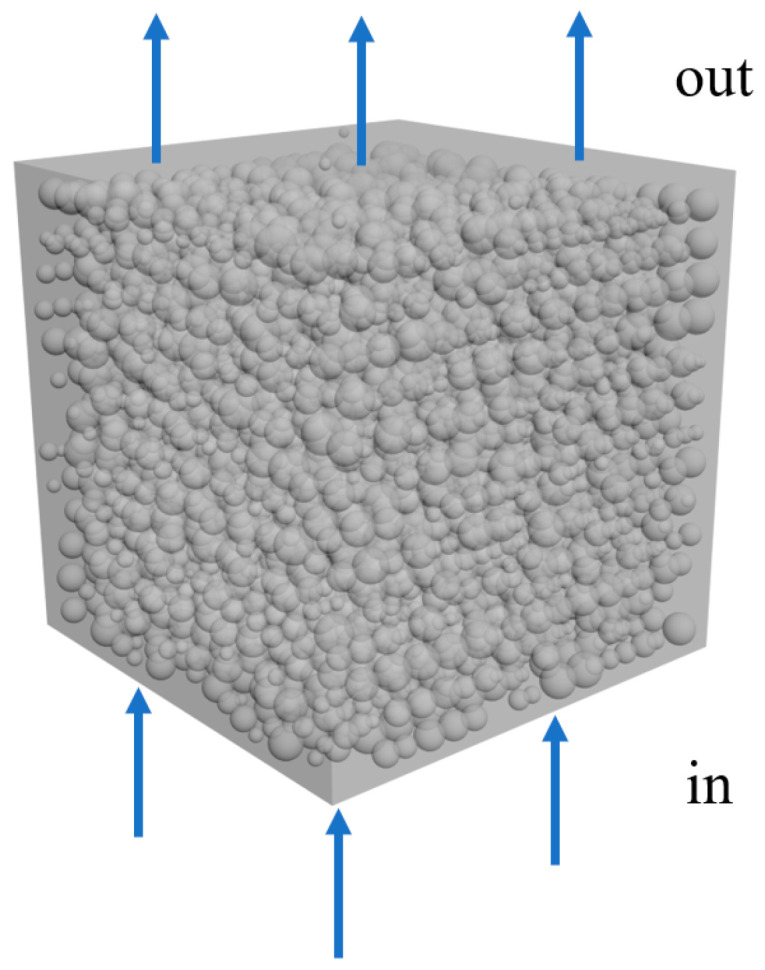
Diagram of flow field.

Suppose the fluid flows in a 4.5×4.5×4.5 mm cube tube, as shown in Figure 8. The fluid is the single-phase flow of a low Reynolds number and is incompressible. The same number of lattice points are used in the x, y and z directions in the grid division of the stacking space. The flow direction of the fluid is in the z direction, the top and bottom surfaces are the fluid outlet and inlet surfaces, respectively, and the other surfaces are boundary surfaces. When the relaxation coefficient is fixed at 1, the universality coefficient is $10^{-6}$. At the beginning, the velocity in the whole flow field is set at 0 and the density is 1. The method to judge the convergence is that in 50 time steps; if the change of the sum of the absolute values of the velocity along the direction of fluid flow is less than 0.0001, convergence is realized.

### 5.4. Simulation Results

#### 5.4.1. The Influence of the Distribution Law

The model is divided into 1283 grid points, and each grid point has 19 velocity directions. The algorithm that is proposed in this paper is used to generate uniform distribution, normal distribution (mean is 3, and the standard deviation is 0.5) and exponential distribution, respectively (rate parameter λ is 2). The simulation results are shown in Table 5.

The above table shows that the average velocity and permeability of exponential distribution are the largest. There is little difference between a uniform distribution and a normal distribution. It should be noted that this example only calculates permeability under different probability density distributions to verify the feasibility of the modeling algorithm. The parameters that are related to the probability density function, such as the direction of the distribution law and the parameters of the distribution function, have significant effects on permeability, and their effects are often coupled with each other. Therefore, further research is needed to establish a general strong law before these questions can be resolved. This modeling algorithm provides a model foundation and technical support for further study of these laws.

#### 5.4.2. The Influence of the Numbers of Particles and Grids

**Figure 9:**
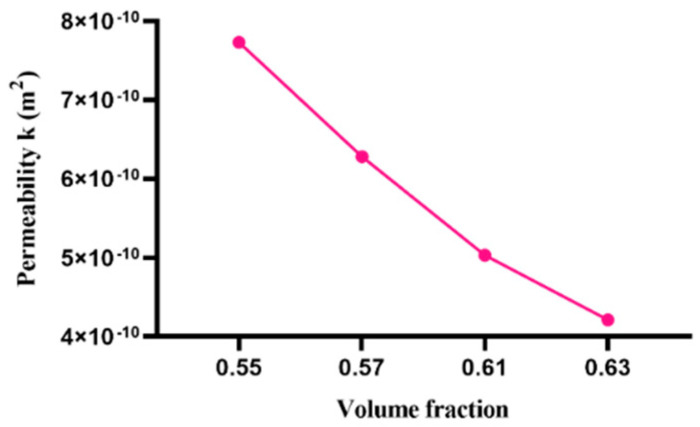
Effect of volume fraction on fluid permeability.

**Figure 10:**
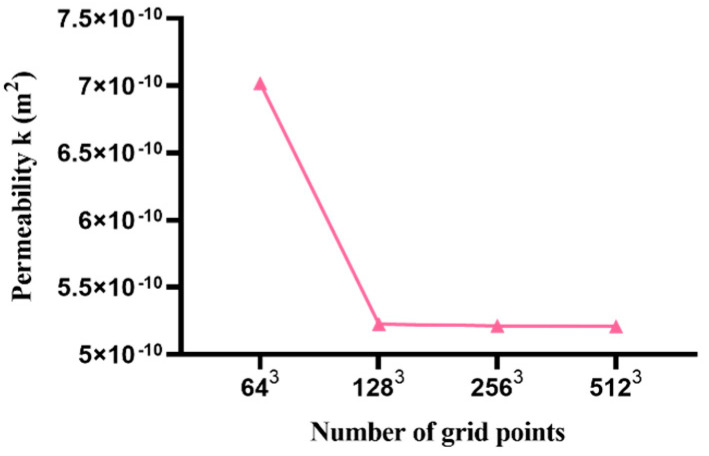
Effect of grid number on fluid permeability.

In the case of the lattice Boltzmann method, both the number of lattice points and the volume fraction of the particles become significant influencing factors. On the premise that the particle distribution law is determined as uniform distribution, the permeability of the model is simulated for the following two cases: (1) the volume fraction of the particles is 0.55 0.57, 0.61,0.63, respectively, and the number of grid points stays the same as 1283; (2) the volume fraction is 0.60 and the number of grid points is 643, 1283, 2563, 5123, respectively. The simulation results are shown in Figure 9 and Figure 10.

Through analytical methods [23], the relationship between the permeability and volume fraction of porous materials can be predicted by specific formulas. The permeability of porous materials is related to the minimum and maximum radius of the cross-section through the transport phase, the shortest path of transport and the volume fraction of the pores. The specific formula is as follows.

$$\hat{\kappa }={\left(0.94\;r_{min}+0.06\;r_{max}\right)}^{2}\;\varepsilon^{2.14}\tau^{-2.44}\;/8\;$$

where $\hat{\kappa }$ is the predicted value of permeability, $r_{min}$ and $\;r_{max}$ are the minimum and maximum radius of the cross-section through the transport phase, ε is the volume fraction of the pores, and τ is the shortest path of transportation.

It can be seen from the formula that the permeability of porous materials increases with the increase in the volume fraction of the pores. It is worth noting that the volume fraction of pore ε and the volume fraction of particle α are complementary, and the sum is 1. This is consistent with the change trend that is shown by the simulation.

As shown in Figure 9, the simulation results show that with the same distribution law, the permeability of the fluid through this distribution decreases with the increase in the volume fraction. Figure 10 shows that under the same distribution law, the permeability of the fluid through the same amount of particle decreases with the increase in the number of lattice points. When the lattice points increase to 1283 or higher, the permeability is almost unchanged.

## 6. Conclusions

In this paper, an efficient algorithm is proposed to generate the particle meso-model by specifying the probability density distribution. The following conclusions and innovations can be obtained:

1. The filling process avoids the huge calculation burden that is caused by the continuous iterative interference detection;
2. In the process of generating the initial position of the particles or realizing the compact stacking, the changes of each particle are only related to its small neighborhood, so it has a high compactness efficiency;
3. The computational complexity of the algorithm is first order, while that of the traditional algorithm is much larger than second order, which illustrates the computational efficiency of the proposed algorithm;
4. With this algorithm, the size and position of the particles can be distributed according to the arbitrary probability density based on the requirements, and the specified volume fraction can also be realized according to the requirements.

The results provide a new method for the space filling and modeling of porous materials and provide technical support for 3D printing when optimizing porous material structures. This method provides a modeling basis for further exploring the influence of porous materials with a specified probability density on the mesoscopic and multi-field simulation research of sound absorption, heat absorption, radiation resistance, seepage and extrusion resistance, which are difficult to observe by experimental methods.

## Figures and Tables

**Table 1:** Distribution effects of particles with different size ranges with the same probability density.

Range of Particle Sizes	The Distribution of Particles
R = 0.05–0.15 mm	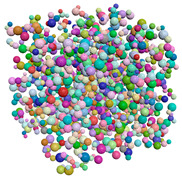
R = 0.15–0.25 mm	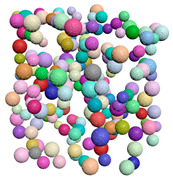
R = 0.25–0.35 mm	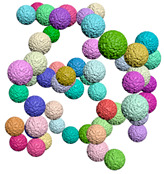

**Table 2:** Distribution effects of particles with the same probability density and different volume fractions.

Volume Fraction	$\mathrm{Total}\;\mathrm{Volume}\;(mm^{3})$	The Distribution of Particles
10%	12.550	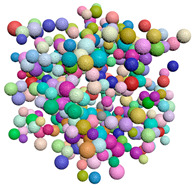
20%	25.504	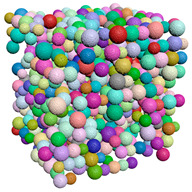
30%	37.743	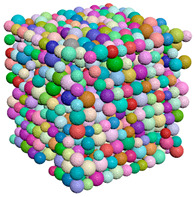

**Table 3:** $\chi^{2}$ test statistical table.

Test Statistics	
Statistic	Value
$\chi^{2}$	11.720
Degrees of freedom	9
Asymptotic significance	0.230

**Table 4:** Normal test analysis results.

Sample Size	Average	Standard Deviation	Partial Degrees	Kurtosis	Kolmogorov–Smirnov Test		Shapiro–Wilk Test	
					The Statistic *D* Value	*p*	The Statistic *W* Value	*p*
301	3.014	0.511	−0.028	−0.219	0.029	0.742	0.996	0.610

**Table 5:** Permeability and average velocity of different distribution.

Distribution	Average Velocity (m/s)	Permeability k $\left(m^{2}\right)$
Uniform	0.586 × 10^−3^	5.204 × 10^−10^
Normal	0.533 × 10^−3^	5.335 × 10^−10^
Exponential	0.681 × 10^−3^	6.757 × 10^−10^

## Data Availability

The data presented in this study are available on request from the corresponding author.

## Symbols and Abbreviations

f	probability density
M	3-dimensional array
ρ	a small positive value
$n_{neighbor}$	a small positive integer
v	volume of the cube
u	probability density
$v_{g}$	total volume of the particles
α	volume fraction
n	particle number
$M_{k}$	number of repeated generation position
$c_{1}$	a positive constant
ϵ	number of all random numbers
g	discrete distribution function
e	velocity space
$\delta_{t}$	discrete time step
t	current time step
Ω	change caused by collision
$C_{s}$	lattice velocity
ω	weight coefficient
$\hat{\sigma }$	fluid velocity
σ	Darcy velocity
h	permeability of porous media
$p_{l}$	fluid pressure
∇	Hamiltonian operator
$\hat{\kappa }\;$	predicted value of permeability
λ	rate parameter of the exponential distribution

## References

[1] Noubactep C., Caré S., Togue-Kamga F., Schöner A., Woafo P. (2010). Extending Service Life of Household Water Filters by Mixing Metallic Iron with Sand. Clean Soil Air Water.

[2] Wang W., Parker K.H. (1995). Effect of deformable porous surface layers on the motion of a sphere in a narrow cylindrical tube. J. Fluid Mech..

[3] Sobieski W. (2021). Waterfall Algorithm as a tool of investigation the geometrical features of granular porous media. Comput. Part. Mech..

[4] Primera J., Hasmy A., Woignier T. (2003). Numerical study of pore sizes distribution in gels. J. Sol-Gel Sci. Technol..

[5] Tory E.M., Cochrane N.A., Waddell S.R. (1986). Anisotropy in Simulated Random Packing of Equal Spheres. Nature.

[6] Jodrey W.S., Tory E.M. (1981). Computer Simulation of Isotropic, Homogeneous, Dense Random Packing of Equal Spheres. Powder Technol..

[7] Cundall P., Strack O. (1980). Discussion: A discrete numerical model for granular assemblies. Geotechnique.

[8] Bezrukov A., Bargiel M., Stoyan D. (2002). Statistical analysis of simulated random packings of spheres. Part. Part. Syst. Charact. Meas. Descr. Part. Prop. Behav. Powders Other Disperse Syst..

[9] Bailakanavar M., Liu Y., Fish J., Zheng Y. (2012). Automated modeling of random inclusion composites. Eng. Comput..

[10] Xin Z., Miao W., Wang Y., Chen H. (2019). Simulation of Tensile Fracture of ZrO2 Toughened Al2O3 Particles Reinforced Fe45 Composites by Finite-Discrete Element Coupling Method. Acta Mater. Sin..

[11] Stevenl B., Peter R.K., David M. (1993). Network Model Evaluation of Permeability and Spatial Correlation in a Real Random Sphere Packing. Transp. Porous Media.

[12] Heijs A.W.J. (1995). Numerical evaluation of the permeability and the Kozeny constant for two types of porous media. Phys. Rev. E.

[13] Bryant S., Blunt M. (1992). Prediction of relative permeability in simple porous media. Phys. Rev. A.

[14] Raats P.A.C. (1973). Dynamics of Fluids in Porous Media. Soil Sci. Soc. Am. J..

[15] Zhu J., Xi Z., Tang H., Tan P. (2006). Characterization of porous structures and fractal theory. Rare Met. Mater. Eng..

[16] Hosmane B.S. (1986). Improved likelihood ratio tests and Pearson chi-squared tests for independence in two dimensional contingency tables. Commun. Stat..

[17] Kline R., Kline R.B., Kline R. (2005). Principles and Practice of Structural Equation Modelling. J. Am. Stat. Assoc..

[18] Drezner Z., Zerom O.T.D. (2010). A Modified Kolmogorov-Smirnov Test for Normality. Commun. Stat.–Simul. Comput..

[19] Lilliefors H.W. (1967). On The Kolmogorov-Smirnov Test for Normality with Mean and Variance Unknown. J. Am. Stat. Assoc..

[20] Bhatnagar P.L., Gross E.P., Krook M. (1953). A model for collision processes in gases: I. Small amplitude processes in charged and neutral one-component systems. Phys. Rev..

[21] Qian Y.H., D‘Humières D., Lallemand P. (1991). Lattice BGK Models for Navier-Stokes Equation. EPL (Europhys. Lett.).

[22] Maier R.S., Kroll D.M., Kutsovsky Y.E., Davis H.T., Bernard R.S. (1998). Simulation of flow through bead packs using the lattice Boltzmann method. Phys. Fluids.

[23] Neumann M., Stenzel O., Willot F., Holzer L., Schmidt V. (2020). Quantifying the influence of microstructure on effective conductivity and permeability: Virtual materials testing. Int. J. Solid Struct..

